# Potentially Pathogenic Airway Bacteria and Neutrophilic Inflammation in Treatment Resistant Severe Asthma

**DOI:** 10.1371/journal.pone.0100645

**Published:** 2014-06-23

**Authors:** Benjamin J. Green, Surasa Wiriyachaiporn, Christopher Grainge, Geraint B. Rogers, Valia Kehagia, Laurie Lau, Mary P. Carroll, Kenneth D. Bruce, Peter H. Howarth

**Affiliations:** 1 Academic Unit of Clinical and Experimental Sciences; NIHR Respiratory Biomedical Research Unit, University of Southampton Faculty of Medicine, Southampton, United Kingdom; 2 Molecular Microbiology Research Laboratory, Pharmaceutical Science Division, King's College London, London, United Kingdom; 3 Department of Respiratory and Sleep Medicine, Hunter Medical Research Institute, John Hunter Hospital, Newcastle, New South Wales, Australia; 4 SAHMRI Infection and Immunity Theme, School of Medicine, Flinders University, Adelaide, Australia; Leiden University Medical Center, Netherlands

## Abstract

**Background:**

Molecular microbiological analysis of airway samples in asthma has demonstrated an altered microbiome in comparison to healthy controls. Such changes may have relevance to treatment-resistant severe asthma, particularly those with neutrophilic airway inflammation, as bacteria might be anticipated to activate the innate immune response, a process that is poorly steroid responsive. An understanding of the relationship between airway bacterial presence and dominance in severe asthma may help direct alternative treatment approaches.

**Objective:**

We aimed to use a culture independent analysis strategy to describe the presence, dominance and abundance of bacterial taxa in induced sputum from treatment resistant severe asthmatics and correlate findings with clinical characteristics and airway inflammatory markers.

**Methods:**

Induced sputum was obtained from 28 stable treatment-resistant severe asthmatics. The samples were divided for supernatant IL-8 measurement, cytospin preparation for differential cell count and Terminal Restriction Fragment Length Polymorphism (T-RFLP) profiling for bacterial community analysis.

**Results:**

In 17/28 patients, the dominant species within the airway bacterial community was *Moraxella catarrhalis* or a member of the *Haemophilus or Streptococcus genera*. Colonisation with these species was associated with longer asthma disease duration (mean (SD) 31.8 years (16.7) vs 15.6 years (8.0), p = 0.008), worse post-bronchodilator percent predicted FEV_1_ (68.0% (24.0) vs 85.5% (19.7), p = 0.025) and higher sputum neutrophil differential cell counts (median (IQR) 80% (67–83) vs 43% (29–67), p = 0.001). Total abundance of these organisms significantly and positively correlated with sputum IL-8 concentration and neutrophil count.

**Conclusions:**

Airway colonisation with potentially pathogenic micro-organisms in asthma is associated with more severe airways obstruction and neutrophilic airway inflammation. This altered colonisation may have a role in the development of an asthma phenotype that responds less well to current asthma therapies.

## Introduction

Although for many years the lower airways were assumed to be sterile, the recent application of molecular microbiological analysis has identified that this is not the case. It is now appreciated that there is a diversity of bacteria present within healthy airways and that this may be disarranged in asthma [1]–[4]. By sampling the lower airways by various methods, including bronchoalveolar lavage [3], protected brushings taken at bronchoscopy [1], [4] and sputum induction [2], the asthmatic airways have been reported to have an altered bacterial microbiota composition, as compared to that in the healthy airways, though there are inconsistencies in the details of these findings. This altered bacterial community composition has been linked to bronchial hyper reactivity in asthma [1] and, more specifically, the presence of *Haemophilus influenzae* has been associated with altered macrophage activation pathways in asthmatics who have relative steroid resistance [3].

Whilst the treatment-resistant severe end of the asthmatic spectrum only represents 5–10% of asthmatics, their disease is associated with disproportionately large healthcare utilisation costs on account of inadequate disease control and frequent disease exacerbations [5], [6].This group, which represents the major unmet clinical need in asthma, is appreciated not to represent a single airway disorder, with induced sputum analysis identifying disease heterogeneity. Both eosinophilic dominant and neutrophilic dominant disease phenotypes are described [7], [8], with neutrophilic disease linked to a sub-optimal response to inhaled corticosteroids [9], [10]. Whilst eosinophilic disease has been associated with T Helper 2 (Th_2_) high disease processes, neutrophilic inflammation may be related to non-Th2 inflammation, such as that associated with activation of the innate immune response [11], [12], as bacteria and bacterial products are recognised to be potent innate immune stimulants. Endotoxins, soluble fragments of lipopolysaccharide (LPS) from the outer membrane of Gram negative bacteria, and Gram positive cell wall components such as lipoteichoic acid (LTA), can act as pathogen associated molecular patterns (PAMPs) which are recognised by Toll-like receptors, CD14 and collectins. Activation of Toll-like receptors leads to an inflammatory cascade resulting in production of the pro-inflammatory cytokines IL-8, IL-1 and tumour necrosis factor alpha (TNF-α), generating a shift toward a Th_1_ and Th_17_ response, neutrophil recruitment and a change in inflammatory cell differential profile [13]. Consistent with this, there is evidence in treatment–resistant severe asthma of increased TNF- α gene and protein expression within the airways compared to that in mild asthma [14]. There has thus been speculation that abnormal airway bacterial colonisation may drive the switch to a neutrophilic phenotype [1]. However, currently there has been no examination of the airway microbiome in a population of severe treatment resistant neutrophilic asthma, or an examination of any differences existing between the microbiota present in patients with severe asthma with different inflammatory phenotypes.

We hypothesised that there would be no difference between the microbiota present in patients with treatment-resistant severe asthma with different underlying inflammatory phenotypes and that airway microbiota would not correlate with clinical measures of disease severity. We therefore used culture-independent Terminal Restriction Fragment Length Polymorphism (T-RFLP) analysis [15]–[17] to profile the bacterial composition of induced sputum samples from treatment-resistant severe asthmatics during a non-exacerbation phase of their disease. We identified numerically dominant bacterial taxa in these infections and assessed their relationship with clinical measures of inflammation and disease. We demonstrate that airway colonisation with *Haemophilus spp., Streptococcus spp.* or *M. catarrhalis* positively correlate with sputum neutrophilia and lower FEV_1_, with *M. catarrhalis* the bacterial species most associated with neutrophilic disease.

## Materials and Methods

### Ethics statement

The study was approved by the Southampton and South West Hampshire Ethics Committee and all subjects provided written, informed consent. Subjects were recruited from the Wessex Severe Asthma Cohort.

### Study design

Asthmatic subjects who had chronic persistent severe disease, as defined by the American Thoracic Society workshop on refractory asthma [18], were selected for study. All participants had disease which fulfilled the following criteria: maintenance treatment with high dose inhaled steroids plus at least 2 add on maintenance therapies, including long acting beta-agonists, leukotriene receptor antagonists and oral steroids at GINA/British Thoracic Society asthma management steps 4 or 5 [19], persistent symptoms (6 part asthma control questionnaire score(ACQ_6_) of > 1.5) that required the need for short acting beta-agonist rescue medication for symptom relief and a history of at least one disease exacerbation within the last year that required a course of oral steroids or an increase in dose of maintenance oral steroids to improve disease control. Alternative causes for symptoms other than asthma had been excluded. No selection restrictions were applied with respect to gender or race. Current smokers, or those who had stopped less than a year previously were excluded. All had been free from an exacerbation for a minimum of six weeks and none were receiving treatment with antibiotics. No patients had clinical or routine immunological evidence of an underlying immune disorder or evidence of clinically relevant bronchiectasis on chest radiographs or lung computed tomography scanning. Detailed clinico-physiological characterisation was undertaken (Table 1).

**Table 1:** Clinical, physiologic and airway inflammation characteristics for subjects with either *Haemophilus sp., Streptococcus sp.*, or *M. catarrhalis* as the most dominant airway species, and subjects with other dominant species.

	*M. catarrhalis*, *Streptococcus* sp. or *Haemophilus* sp. dominant	Other dominant species	Significance
Subjects	17	11	
Sex, M/F	6/11	1/10	0.191
Age∧ [years]	51.7 (29–67)	41.6 (19–76)	0.083
BMI	28.3 (4.6)	27.1 (6.6)	0.499
Ex-smokers	8	6	0.699
Smoking pack years∧	3.0 (0–30)	6.7 (0–28)	0.073
Maintenance oral prednisolone	9	4	0.39
Prednisolone mg/day*	8.4 (9.4)	6.3 (8.4)	0.623
ICS (BDP mcg Eq/day)*	2357 (936)	1927 (671)	0.285
Late onset asthma (after12)	12	6	0.444
Atopic	12	6	0.444
FEV_1_ % predicted post-bronchodilator*	68.0 (24.0)	85.5 (19.7)	0.025**
Percent reversibility*	5.5 (8.4)	8.8 (12.1)	0.664
PEFR Variability %*	30.0 (17.0)	18.0 (12.4)	0.268
Duration of asthma [years]*	31.8 (16.7)	15.6 (8.0)	0.008**
Severe exacerbations last 12 months median (IQR)	4 (3–6)	2 (1–6)	0.29
ACQ Score*	3.03 (1.2)	2.87 (0.88)	0.723
Exhaled NO [ppb]*	12.5 (9.4)	12.5 (8.5)	0.851
Median % Neutrophil count (IQR)	80 (67–83)	43 (29–67)	0.001**
Neutrophil count >61%	13	4	0.008**
IL-8 concentration, pg/ml [Median (IQR)]	5192 (9805)	1315 (2221)	0.08

* Values are mean (SD), ∧ Values are mean (Range), ** Significant with p<0.05. BMI – body mass index, ICS – inhaled corticosteroid, BDP – beclomethasone dipropionate, Eq – equivalent, FEV_1_ – forced expiratory volume in 1 second, PEFR – peak expiratory flow rate, ACQ – Asthma Control Questionnaire, NO – nitric oxide, IL8 – interleukin 8.

### Sample collection and processing

Sputum was induced using hypertonic saline and collected in accordance with the European Respiratory Society guidelines. If participants failed to produce sputum, they were excluded from the study, all sputum samples collected were analysed and the data presented [20]. Sputum plugs were selected from expectorates and divided for differential cell counting with the remainder stored at −80°C for subsequent molecular analyses. Sputa for differential cell counts were immediately processed with dithioerythritol, to separate cells from the fluid phase, homogenised, filtered using a 100 µm cell strainer (BD Falcon) to remove mucus, centrifuged at 10 G, the supernatant removed and the cell pellet resuspended in phosphate buffered saline. A total cell count was made using a haemocytometer and cell viability determined using trypan blue exclusion.

Cytospins were prepared using 7×10^4^ cells and centrifuged at 450 rpm for 6 minutes (Shandon Cytospin 2). Cytospins were fixed with and stained using Rapi-Diff I, II and III (DiaCheM Int. Ltd, UK) and differential cell counts were obtained from 400 non-squamous cells.

### Sputum Supernatant and IL-8 Measurement

Interleukin-8 measurements were undertaken on the sputum supernatant using the CXCL8/IL8 DuoSet ELISA kit (R&D Systems, UK) as per the manufacturer's instructions.

### Terminal Restriction Fragment Length Polymorphism (T-RFLP) Profiling

Sputum samples were processed for T-RFLP analysis as described previously [16]. Briefly, nucleic acid was extracted directly from induced sputum samples and a 927 base fragment of the 16S rRNA genes amplified with a 5′ IRD700-tagged primer. Subsequently, the amplified 16S rRNA genes were digested with *Cfo*I and resolved on a IR2 automated DNA sequencer (LI-COR Biosciences). T-RFLP profiles were analysed using Phoretix one-dimensional Advanced software, v.5.10 (Nonlinear Dynamics, Newcastle-upon-Tyne, UK). T-RF band sizes were determined by comparison with MicroSTEP-15a (700 nm) size marker (Microzone, Lewes, UK). T-RF band volume was determined and expressed as a percentage of the total volume of bands detected in each electrophoretic profile. These percentages are reported here as “relative abundance” T-RF bands from each sample profile were rank ordered, with bacterial species from which the highest percentage relative abundance band was derived classified as “dominant” within the airway microbiota.

### Asthma Sub-typing

Subjects were defined as having neutrophilic asthma if the sputum neutrophil differential cell count was greater than 61% of non-squamous cells.

### Statistics

Data were analysed using SPSS Statistics (IBM, New York, USA). Non-normally distributed data were compared using the Mann-Whitney U-test. Correlations between nonparametric data were undertaken using Spearman's rank correlation. Two-tailed tests were used and the level of significance was taken as p = 0.05.

## Results

Induced sputum was collected from 28 severe asthmatics for T-RFLP analysis (Table 1). These comprised 7 men and 21 women with a mean age of 47.7 yrs (range 19–75) and a mean (±SD) disease duration of 25.4 (±15.9) years. Fourteen subjects were life-long non-smokers and 14 ex-smokers. Their mean post-bronchodilator FEV_1_ (±SD) was 74.8±23.6 percent predicted and they were all receiving high dose inhaled steroids with a mean daily dose equivalent to 2189 µg of inhaled beclometasone. In addition, 13 subjects were receiving long-term maintenance therapy with oral steroids. Eighteen subjects were atopic, 18 had disease of late onset, as defined by onset after the age of 12 years, and all had inadequate disease control (ACQ_6_>1.5) with a mean ACQ_6_ score of 2.96 (±1.1). In 2 of these 28 volunteers the sputum sample size was insufficient to obtain a reliable differential cell count. The appropriateness of the sputum samples obtained in the others was indicated by a mean total differential cell squamous cell counts of 8.1% (±11.5). Seventeen of these treatment-resistant severe asthmatics had neutrophilic asthma.

16S rRNA gene T-RFLP analysis was used to profile the total airway bacterial community in each sample. Whilst diverse, samples analysed here were typically dominated by one bacterial species that represented a majority of total bacterial abundance. We therefore assessed the relationship between the identity of this predominant species and clinical measures of disease, a technique that has been used successfully to identify relationships between infective microbes and disease course in non-cystic fibrosis bronchiectasis [21].

**Figure 1:**
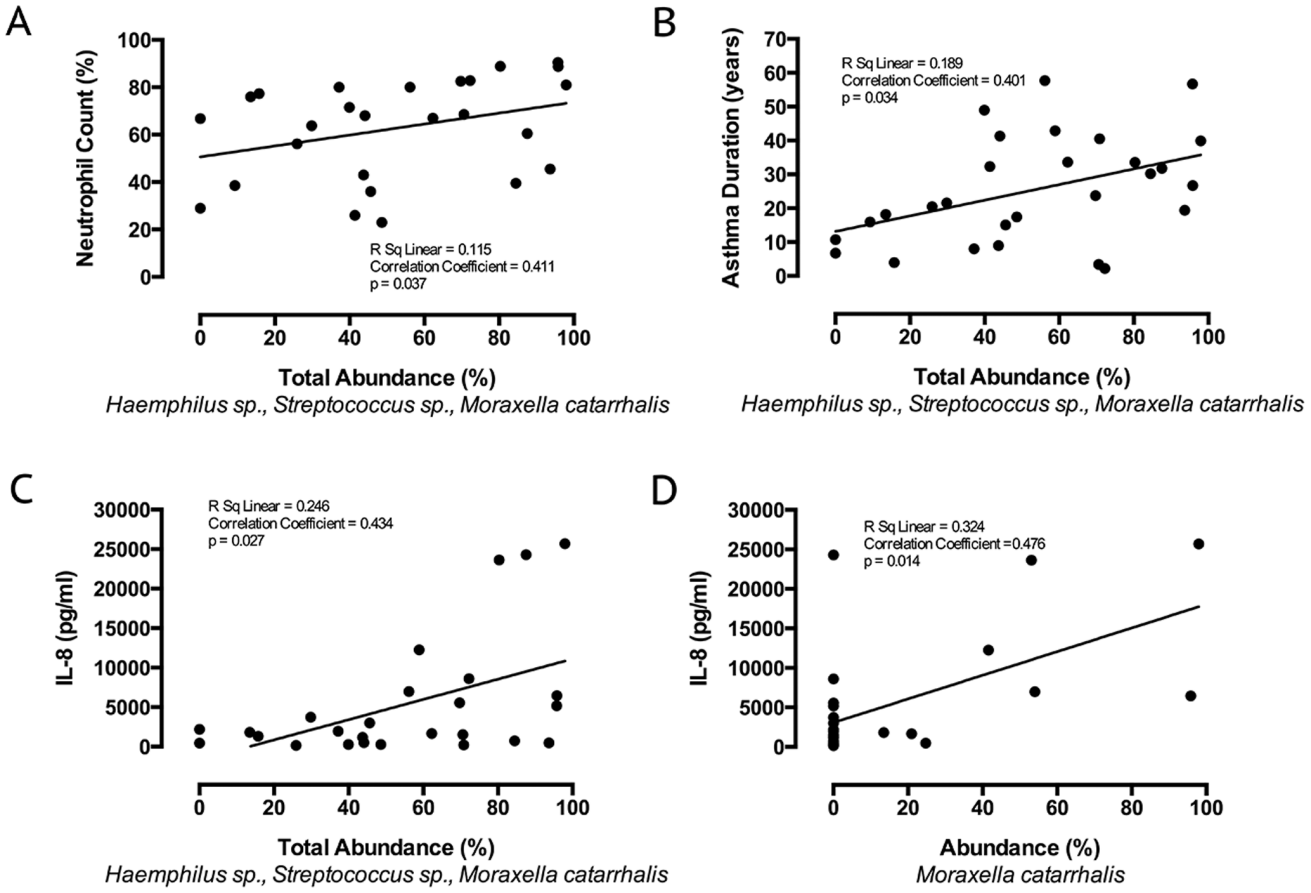
Severe asthma disease duration and inflammation related to abundance of *Haemophilus., Streptococcus*, and *Moraxella sp.* The relationship in treatment-resistant severe asthma between total abundance of *Haemophilus sp., Streptococcus sp.*, and *Moraxella catarrhalis* in induced sputum samples and [A] neutrophil differential cell count (%), [B] asthma duration (years), [C] interleukin (IL)-8 concentration in induced sputum and [D] the relationship between *M. catarrhalis* abundance and induced sputum IL-8 concentration.

The potentially pathogenic micro-organisms (PPMs), *Haemophilus sp., Streptococcus sp.*, or *M. catarrhalis* were identified as the numerically dominant species in the majority (17/28) of patients (Table 2). Other dominant taxa (number of subjects) were, *Veillonella sp.* (5), *Neisseria sp*. (2), *Prevotella sp*. (2), *Pseudomonas aeruginosa* (1) and an unassigned species (1). The presence of these defined PPMs - *Haemophilus sp., Streptococcus sp.*, or *M. catarrhalis* - as a dominant species was found to be significantly associated with neutrophilic asthma (p = 0.008, Table 1). Dominant airway colonisation with any of these three bacteria taxa was associated with a higher neutrophil differential cell count (Median (IQR) 80% (67–83) vs 43% (29–67), p = 0.001, Table 1) and the relative abundance of *Haemophilus sp., Streptococcus sp.*, and *M. catarrhalis* within sputum samples was positively correlated with the neutrophil differential cell count (p = 0.037, Figure 1).

**Table 2:** Number of subjects and mean percentage abundance of different bacterial species present within induced sputum samples.

Predominant taxon	Subjects	Mean percentage relative abundance (range)
*Haemophilus* sp.	6	63.9 (26.5–95.8)
*Streptococcus* sp.	6	47.2 (25.6–74.6)
*M. catarrhalis*	5	68.5 (41.6–98.0)
*Veillonella* sp.	5	43.8 (27.5–80.2)
*Neisseria* sp.	2	41.0 (39.6–42.3)
*Prevotella* sp.	2	30.5 (23.8–37.1)
Unassigned species	1	36.5
*P. aeruginosa*	1	48.8

**Figure 2:**
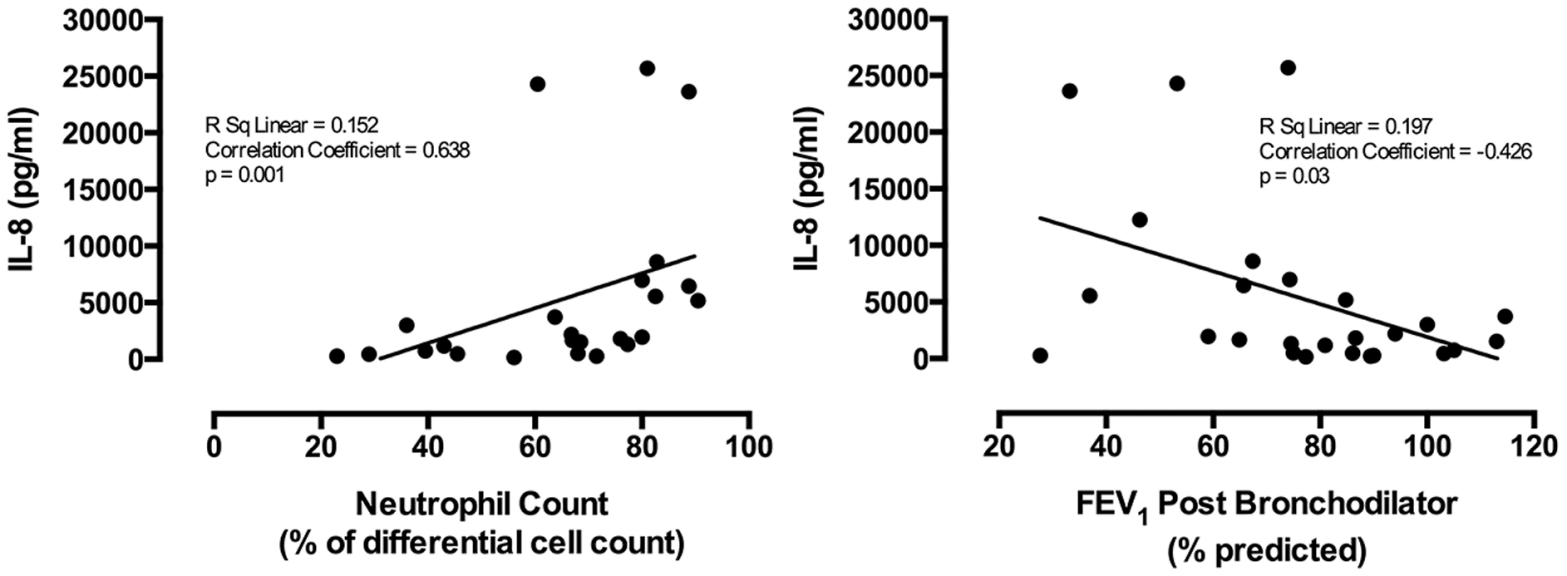
Interleukin-8 sputum supernatant concentrations in severe asthma related to sputum neutrophil count and lung function. The relationship in treatment-resistant severe asthma between IL-8 concentrations in induced sputum and (A) neutrophil differential cell counts and (B) Post bronchodilator percent predicted FEV_1_.

In subjects where *Haemophilus sp., Streptococcus sp.*, or *M. catarrhalis* predominated within airway microbiota, post-bronchodilator percent predicted FEV_1_ was significantly lower than in subjects where any other bacterial species was dominant (Mean (SD) 68.0% (24.0) vs 85.5% (19.7), p = 0.025, Table 1). Dominance of these species was also associated with longer disease duration (31.8 years (16.7) vs 15.6 years (8.0), p = 0.008) and relative abundance positively correlated with disease duration (p = 0.037, Figure 2). Individually, *M. catarrhalis* was the bacterial species most associated with sputum neutrophilia, with a mean sputum neutrophil count of 84.2% (±4.3) in the 6 patients where it was predominant, compared to 57.6% (±20.1) in subjects where it was not.

Sputum IL-8 concentration was determined in the 26 samples where sufficient material was available. Sputum IL-8 concentration was higher where *Haemophilus sp., Streptococcus sp.*, or *M. catarrhalis was* dominant compared to subjects where another taxon was dominant, although this did not reach standard statistical significance (p = 0.08, Table 1). Sputum IL-8 concentration did, however, correlate significantly with the total abundance of *Haemophilus sp., Streptococcus sp.*, and *M. catarrhalis* (r = 0.43, p = 0.027, Figure 1). The strongest species correlation with sputum IL-8 concentrations was with *M. catarrhalis* (r = 0.48, p = 0.014). Sputum IL-8 also correlated with neutrophil count (r = 0.68, p = 0.001, Figure 2) and lower FEV_1_ post bronchodilator (r =  0.43, p = 0.03, Figure 2). Raw data tables are available in supporting information file Data S1.

## Discussion

We demonstrate that potentially pathogenic bacteria are present within the airways in severe asthma and that the predominance of *M. catarrhalis* or members of the *Haemophilus or Streptococcus* genera is associated with a neutrophilic airway phenotype in treatment-resistant persistent asthma. These microorganisms have been associated with an increased risk of wheeze and early life asthma, when present in neonates [22], and with worse and progressive chronic obstructive pulmonary disease (COPD) in cigarette smokers [23]. Our findings in severe asthma, demonstrating the relevance of altered airway colonisation with pathogenic bacteria, are thus concordant with previous data in other airway diseases.

In stable, smoking-related COPD, the presence of these bacterial taxa within the lower airway is associated with an increase in absolute neutrophil counts [23] as well as higher concentrations of the neutrophil chemoattractant IL-8 [24], TNF-α [25], and endotoxin [23]. PPM detection in COPD is also associated with worse clinical outcomes including health status [25], exacerbation rates [26] and rate of FEV_1_ decline [24]. In our study examining asthma, the identification of these species within sputum samples was associated with significantly lower lung function. In addition these species were associated with a non-significant increase in exacerbation rate in the preceding 12 months. There is thus consistency with the findings from COPD, suggesting that the findings in treatment-resistant severe asthma could have relevance to an adverse clinical outcome and provide an explanation for the inadequate response to standard asthma therapy in this severe asthma group. In our study in asthma, none of the subjects were current smokers although fourteen were ex-smokers. There was, however, no difference between the life-long non-smokers and the ex-smokers in their profile of bacterial dominance or their sputum neutrophil counts to indicate the relevance of cigarette smoking history to these findings in severe asthma, it is possible, however that this lack of difference could be explained by the small sample size examined here.

Non-eosinophilic, neutrophilic asthma represents up to 25% of symptomatic asthma patients and 59% of patients on high dose inhaled corticosteroids attending asthma clinics [10], [12]. Whilst asthma is considered predominantly a T Helper 2 (Th_2_) lymphocyte driven disease, with Th_2_ cytokines driving eosinophilic inflammation, innate immune mechanisms may lead to a shift towards Th_1_ or Th_17_ mediated neutrophilic inflammation in a significant group of patients with severe asthma. Increased airway bacterial load and specific bacterial species will lead to increased soluble bacterial cell wall fragments such as LPS from the outer cell membrane of Gram negative bacteria within the airway. The lipid portion of the molecule, unlike the polysaccharide, is not antigenically specific and is able to bind to pattern recognition receptors to trigger the innate response and neutrophil recruitment. Previous studies in COPD have shown that *Haemophilus sp., Streptococcus sp.*, and *M. catarrhalis* are associated with increased markers of innate immune activation and there is biological plausibility that these species may have a role in neutrophilic asthma. Consistent with this is a previous study in chronic persistent severe asthma (n = 9) which demonstrated increased mRNA expression of the innate immune receptors TLR2, TLR4 and CD14, as well as the cytokines IL-8 and IL-1β in induced sputum from patients with neutrophilic asthma [11]. In this study, neutrophilic airways inflammation was associated with higher airway LPS and potentially pathogenic bacteria were cultured in 43% of patients. Although previous culture independent microbiology studies on the asthmatic airway have not reported any differences in microbiota composition between inflammatory phenotypes, these studies were not designed to examine severe treatment-resistant disease [1], [4]. Although there was no statistically significant difference in the doses of oral or inhaled corticosteroids between the groups in our study based on microbiota composition, there was a trend towards a difference. This may reflect a worse disease phenotype caused by potentially pathogenic bacteria, or the higher steroid doses could have induced a selection pressure within the airway. Currently it is unclear which, if either, of these mechanisms is most important; prospective studies may provide an answer in due course.

Induced sputum is a well-recognised technique for examining lower airway inflammation. Further, the suitability of induced sputum as a basis for analysis of airway microbiota has been demonstrated previously [27], [28]. Although expectorated sputum passes the oropharynx, which raises the potential for upper airway contamination, it has been established that if there is low salivary contamination of the sputum sample, as reflected by fewer than 25% squamous cells, that sputum is a reliable sample for analysis as cultured bacterial species resemble those from a transtracheal aspirate [29] and that contamination of sputum with bacteria originating in the oral cavity is relatively low [30]. Similarly in COPD it has been demonstrated using culture independent techniques that sputum and bronchial aspirate samples are similar to each other [31]. As the sputum samples analysed in our study had a mean squamous cell count of 8.1%, these previous findings would be supportive of their use in evaluating lower airway bacterial colonisation.

If the airway microbiome is relevant in neutrophilic asthma, it would be expected that antibiotic therapy might be of benefit. Whilst studies of chronic stable asthma, undifferentiated by inflammatory phenotype, have been disappointing [32], two studies selecting or analysing by inflammatory phenotype have shown encouraging results. In one, clarithromycin given orally for 8 weeks was reported to improve asthma quality of life scores and reduce airway IL-8 in patients with non-eosinophilic airway inflammation when compared to those with eosinophilic disease [33]. In the other, adding azithromycin to standard therapy in exacerbation prone asthma was only effective in reducing the rate of severe exacerbations and lower respiratory tract infection in patients with non-eosinophilic disease [34]. These studies provide clinical support to our data suggesting that the airway microbiota in non-eosinophilic (neutrophilic) asthma is different to eosinophilic phenotypes, and may be manipulated to improve clinical outcomes.

In using the identity of the numerically dominant bacterial taxon as a basis for assessing relationships between airways microbiology and disease, our aim was to focus on infective populations most likely to give rise to altered inflammatory responses; a strategy that has been applied successfully in other respiratory contexts [21]. Assessing whether the composition of the airway microbiota excluding the dominant species is distinct in severe asthma was not performed here, but represents a further area where important insight might be gained. A further important area for investigation is the extent to which the relationships between PPM predominance and disease severity reported here might be causal, a question that might be addressed through prospective longitudinal analysis within individual subjects.

The present study provides evidence to implicate the presence of airway pathogenic bacteria, in particular *Haemophilus sp., Streptococcus sp.* or *Moraxella catarrhalis* in neutrophilic treatment-resistant severe asthma. Sputum IL-8 concentrations correlated with the total abundance of PPMs, particularly *Moraxella catarrhalis*, linking disease pathophysiology with this abnormal airway colonisation. As asthma is a heterogenous disease, with neutrophilic asthma being recognised to represent a more steroid resistant phenotype, a greater understanding of the molecular mechanisms involved in the development of this phenotype may enable the development of improved targeted therapies that reduce the airway colonisation and the abnormal airway inflammatory response associated with this altered pathogenic bacterial load.

## Supporting Information

Data S1 (XLSX)
